# Vindication of Mr. Davy's Discoveries

**Published:** 1809-02

**Authors:** 


### *To the Editors of the Medical and Physical Journal.*

GENTLEMEN,

I Was much pleased on your first announcing a new Editor, to find your determination of avoiding all personal remarks to the disadvantage of another. It is not my intention to accuse you of any deviation from your promised plan, but merely to remark, by how many oblique ways you may be deceived whilst you are fancying yourselves pursuing the direct road. The paper in your last, signed "JACOBUS," is a remarkable instance of this; serving not only as a means of *indirectly* lessening the merit of one person, but of directly *exalting* another.

Though my chemistry is almost as old as Priestley's, yet I contrive, as well as I can, to keep up some knowledge of what is going on in the 'laboratories. Among other things, it was impossible not to be struck with some late discoveries, or not to be amused with the different manner in which they have been received. Nothing, however, has entertained me so much as Jacobus's letter.

"In the days of Van Helmont and Becher!" What? "It was doubted whether earths were simple bodies." This doubt was further increased when Scheel had discovered pyrites, and when Lavoisier had shown that metals, by the addition of oxygen, assumed an earthy or pulverulent appearance. Then it was that Tondi and Ruprecht fancied they had discovered metallic buttons by reducing earths with charcoal. But Klapstock and Savaresi showed they were wrong.

"Hitherto," continues Jacobus, "we have seen that earths *only* were suspected to be of a compound nature. That idea was never extended to alkalis, until some years afterwards. The reasons that existed for cherishing a suspicion of the sort, with regard to the latter class of bodies, were the alkaline nature of barytes, magnesia, and lime; and although these were taken no notice of by the foreign chemists, they did not escape the observation of an acute countryman of our own, Mr. Kerr, a short time afterwards. This gentleman was the translator of Lavoisier's Elements; and to that part of the work, where Lavosier states his conjectures with regard to the earths, Mr. Kerr added an account of the experiments of Von Ruprecht, Tondi, and Born. This he accompanied with comments; and seizing the idea of the alkaline nature of four four of the earths (for strontian had, at this date, been added by Dr. Hope) he developed from a fine train of reasoning, the opinion that "all bodies in nature may be divided into oxygen, inflammable matter, and caloric;" that "*alkalinity* and acidity depended upon the doses of oxygen with which the combustible was combined;" and, in fine, that "*the alkalies were* metallic substances *in some hitherto unknown state of combination*."

Now, Gentlemen, give me leave to put this into different language, without in the least disputing a single fact offered by your correspondent.

That chemists, in the dawn of science, should doubt the elementary properties of earth, can surely be no matter of wonder, when they must have seen the decomposition of their own furnaces. Whether Tondi and the rest, really produced buttons of sufficient magnitude to justify their doubts, it is hardly worth while to inquire; for as they made their attempts with charcoal, is it now unnecessary to produce the authority of Klapstock and Savaresi that they were wrong? Mr. Davy has shown that the bases of earths and alkalis attract oxygen infinitely more powerfully than charcoal, consequently that no metal could have been reduced from them in this way. But when Mr. Kerr translated Lavoisier, this fact was not known. The translator, therefore, thought there could be no harm in reasoning on the buttons of Tondi, as if they were really produced from earths. "To that part of the work," says Jacobus, "where Lavoisier states his conjectures with regard to earths, Mr. Kerr added an account of the (as he before remarks, and as it is now confirmed erroneous) experiments of Von Ruprecht, Tondi and Born;" that is, if I understand him rightly, the experiments of these gentlemen, though unsatisfactory to the accurate Klaproth, might serve Mr. Kerr to reason upon; "and seizing the idea of the alkaline nature of four of the earths," he guessed, by good luck rightly, that if these were metals, all the alkalis might be so likewise.

Now mark, Gentlemen, from this event, the ingenious manner in which an attempt is made to exalt the translator of Lavoisier at the expence of Davy, who, we are told, "has rendered himself immotral;" to whom "is to be given the first praise;" "whose fostering genius has kindled the discoveries of Lavoisier and Kerr into a flame, after the pile had been prepared by Volta."

Αλλα με μοιρ' ολοη, και Λητους εκτανεν υιος,
Ανδρων δ' Ευφορβος' συ δε με τριτος εξεναριζεις

*Homer*, Π, 849.

But the language of Jacobus to "this immortal" "fostering genius," to whom "the first place should be given," is infinitely more contemptuous than any expression from Patroclus. Hector, at least, gave the *coup-de-grace,* but Davy has done no more than light a fire constructed by Volta for conducting a process instituted by Kerr.

After all, gentlemen, what is the merit of this discovery, of which we hear so much, and the result of which it is impossible to estimate? Mr. Davy, if he feels as I should do, must be mortified to hear so much about a single result of a set of laws, which he discovered, as all physical laws must be discovered, by reasoning on the result of a number of facts. Those who have followed him through his courses, will recollect when he first stated in his Lectures on Chemical Electricity—That all bodies of known composition, when attracted by the negative surface of the Voltaic circle, consist principally of inflammable matter. The probable inference was, that bodies whose composition was unknown, if attracted by the same surface, would ultimately be found to consist principally of inflammable matter also. Finding the alkalis attracted by the negative surface, he endeavoured to discover some inflammable principle in them, and to his own astonishment discovered a metal. Thus what was guessed from the fallacious experiments of Ruprecht and others, was proved to be true; an event by no means uncommon in the most usual transactions of life.

It is unnecessary to dwell on this subject any longer, than to request *Jacobus,* instead of applauding the hardy guesses of others, calmly to pursue a few philosophical inquiries, either with his own experiments, or with such as are well ascertained by others. He will then know the value of researches conducted with accuracy and with a certain object in view. I shall be glad to know what Mr. Kerr has done in this way, as it is very much my wish that every one should have the merit of his own talents and labour? For this reason, I cannot help expressing my surprize, that Jacobus should give Lavoisier the merit of discovering oxygen. That philosopher has merits enough of his own, without detracting any thing from others, particularly from that original experimenter Priestley, to whom we undoubtedly are indebted for the discovery of oxygen.

JOANNES.

*January* 7, 1809.

